# Organizational principles of multidimensional predictions in human auditory attention

**DOI:** 10.1038/s41598-018-31878-5

**Published:** 2018-09-07

**Authors:** Indiana Wollman, Benjamin Morillon

**Affiliations:** 1 Montreal Neurological Institute, McGill University, Montreal, Canada; 2 CIRMMT, Schulich School of Music, McGill University, Montreal, Canada; 3 Aix Marseille Univ, Inserm, INS, Inst Neurosci Syst, Marseille, France

## Abstract

Anticipating the future rests upon our ability to exploit contextual cues and to formulate valid internal models or predictions. It is currently unknown how multiple predictions combine to bias perceptual information processing, and in particular whether this is determined by physiological constraints, behavioral relevance (task demands), or past knowledge (perceptual expertise). In a series of behavioral auditory experiments involving musical experts and non-musicians, we investigated the respective and combined contribution of temporal and spectral predictions in multiple detection tasks. We show that temporal and spectral predictions alone systematically increase perceptual sensitivity, independently of task demands or expertise. When combined, however, spectral predictions benefit more to non-musicians and dominate over temporal ones, and the extent of the spectrotemporal synergistic interaction depends on task demands. This suggests that the hierarchy of dominance primarily reflects the tonotopic organization of the auditory system and that expertise or attention only have a secondary modulatory influence.

## Introduction

Prominent theories in neuroscience assume that brain neural activity is shaped by one’s perceptual, behavioral and emotional experiences and reflects historically informed internal models of causal dynamics in the world, that serve to generate predictions of future sensory events^1–3^. However, it remains largely unknown how predictions are encoded in the brain in particular because they are essentially multidimensional. Indeed an event can be anticipated with regard to its features (content), location (space), and moment of occurrence (time), by exploiting contextual regularities in the environment. If properly predicting each of these dimensions has been shown to optimize performance in the visual domain^4,5^, the organizational principles governing how multiple types of predictions combine remains unclear. Several studies revealed that they do not combine in a linear (or additive) fashion, but rather synergistically interact to optimize sensory processing^6–14^ (but see^15^). This indicates that neural systems coding for different dimensions of the environment do not operate in parallel to optimize perception. Rather, multidimensional predictions are mediated through a processing bottleneck^12^, with some dimensions dominating – or being prioritized – over the others, supporting a *hierarchy of predictive filters* in sensory cortices.

Two views can be put forth to explain what governs this hierarchy. The hierarchy of dominance could reflect the physiological constraints imposed by the topography of the sensory systems of interest. In the visual and auditory systems, retinotopic and tonotopic organizations are respectively preserved throughout the hierarchy of sensory processing stages, allowing for a common reference frame centered around one dimensional feature space^16,17^. Further, the validity of spatial^11^ and spectral^14^ predictions were respectively suggested to condition the effectiveness of temporal predictions, these latters exerting an influence in a receptive field-based manner^18^. On the other side, the effect of one-dimensional predictions on sensory processing depends on attention^19–21^. Attentional priority is internally represented as a hierarchical structure that dynamically adjusts to reflect task demands (i.e. behavioral relevance^22,23^, its mechanistic role being to weight or bias the processing of selected attributes of a stimulus^24^. Therefore, the hierarchy of dominance could also reflect attention that flexibly prioritizes behaviorally relevant dimensions over non-relevant ones.

In the present study, we conducted six behavioral experiments to disentangle if priors belonging to different dimensions interact according to physiological constraints or flexibly combine as a function of behavioral relevance or past knowledge. Not only did we manipulate attentional demands across experiments but we also conducted cross-sectional experiments involving groups of participants differing only in their long-term perceptual skill training. Perceptual expertise has indeed been demonstrated to shape and enhance perceptual and cognitive skills^25–27^, and musical expertise, in particular, has been suggested to rely on greater prediction skills^28–30^. Hence, we placed our investigation in the auditory domain where temporal and spectral dimensions are essential^31^, and conducted the same psychophysical experiments on two groups of participants: musical experts (musicians) and non-musicians. While the effects of temporal predictions on behavioral performance have been extensively studied (e.g.^14,32,33^, the effects of spectral predictions are still scarce^9,14,15^. Even more so, whether the impact of one- or two-dimensional(s) predictions on perceptual sensitivity depends on behavioral relevance or perceptual expertise remains unknown.

Here, we reasoned that if multidimensional predictions are organized according to a fixed canonical model (physiological constraints or attentional priority), we should not observe *qualitative* difference between the two groups of musical expertise in their ability to exploit and combine predictions. In other words, we should not observe any change in the hierarchical organization of predictive filters between groups, only quantitative (or no) difference in their ability to exploit predictions. We first assessed the respective role of temporal and spectral predictions in deviant detection tasks involving either temporal or spectral attention in both musicians and non-musicians. Then, we examined how priors belonging to temporal *and* spectral dimensions combine and whether this depends on behavioral relevance or perceptual expertise.

## Results

In this study, participants underwent six psychophysical experiments. By presenting auditory sequences of pure tones with either no regularity or high regularity on their spectral and/or temporal dimension (hereafter called respectively “unpredictable condition” or “predictable condition”, with “predictability context” referring to this manipulation; see Method), we investigated whether prior information influenced participants’ ability to detect deviants presented at their perceptual threshold level (difficulty was thus titrated individually; see Method), and diverging from the standards either in their temporal or spectral dimension. It is important to note that during the proper experiment, we were not interested in participants’ ability to detect deviant tones in one task but in how participants’ performance varies across “predictable condition” and “unpredictable condition” within that particular task (deviants’ properties relative to the background stream being similar across conditions).

In the first four experiments (exp. 1–4), we studied the effect of each type of prediction in isolation (i.e. spectral only or temporal only) in perceptual detection task requiring either spectral attention or temporal attention. In the two last experiments (exp. 5–6), we studied the interaction between these two sources of predictions and compared their effect when they are provided in spectral versus temporal tasks. Moreover, in all experiments we investigated the potential influence of musical expertise on auditory deviant detection. Hence, two groups of participants, musical experts and non-musicians, took part in all six experiments.

### One-dimensional predictions optimize perceptual sensitivity independently of task-demands and musical expertise

**Figure 1:**
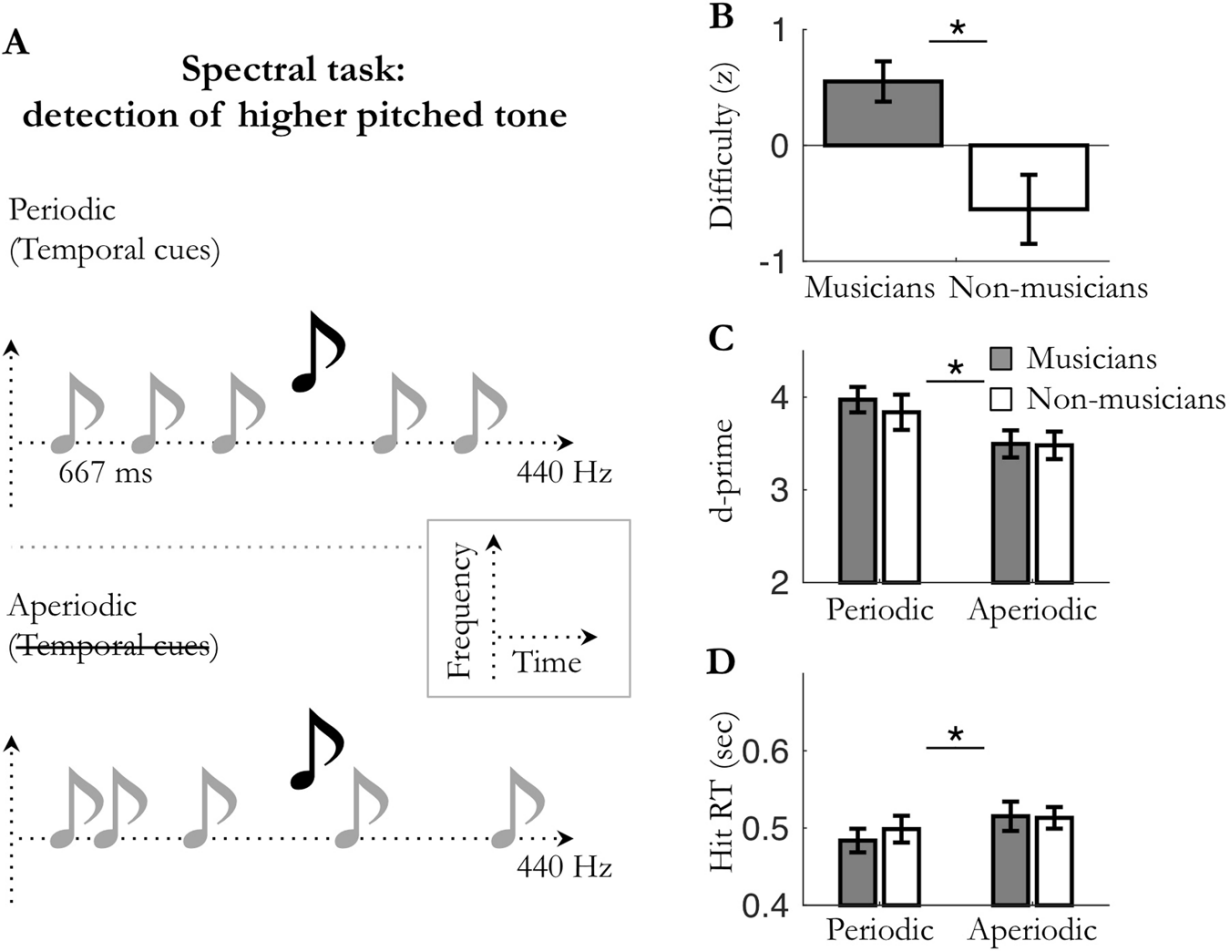
Experiment 1: spectral attention in *periodic*/*aperiodic* monotone conditions. (**A**) Monotone (e.g. 440 Hz) auditory streams of pure tones were presented in the *periodic* (fixed SOA; 667 ms) or the *aperiodic* (jittered SOA) condition. Participants had to detect spectrally deviant, higher pitched tones. (**B**) Averaged difficulty for musicians and non-musicians. Difficulty was individually tailored to equalize performance accuracy across participants. (**C**) Averaged sensitivity (d′) and (**D**) hit reaction times. Stars indicate significant differences (n_mus_ = 12; n_n-mus_ = 12; *p* < 0.05).

**Figure 2:**
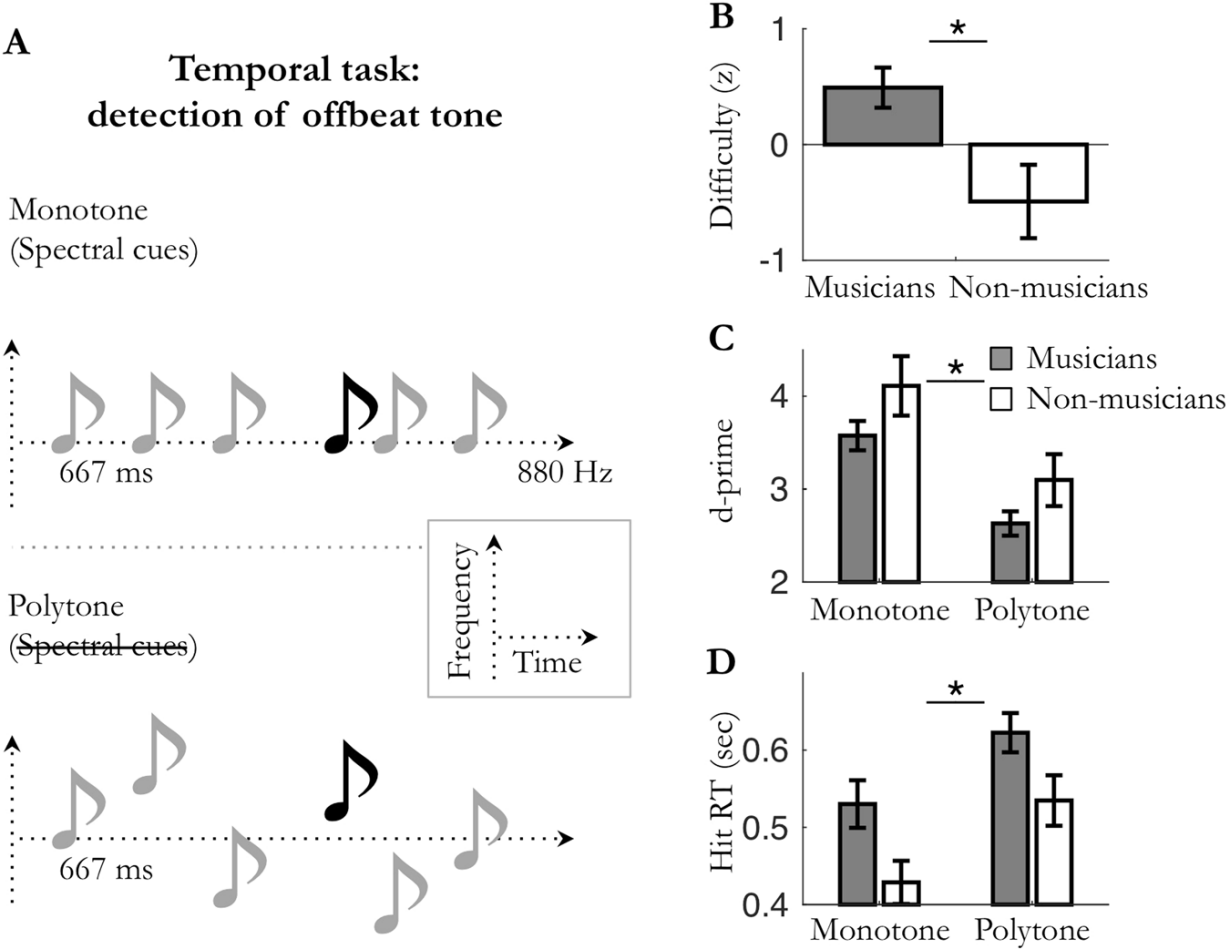
Experiment 2: temporal attention in *monotone*/*polytone* periodic conditions. (**A**) Periodic (1.5 Hz) auditory streams of pure tones were presented in the *monotone* (fixed pitch; e.g. 880 Hz) or the *polytone* (jittered pitched) condition. Participants had to detect temporally deviant, offbeat tones. (**B**) Averaged difficulty for musicians and non-musicians (set to equalize performance accuracy). (**C**) Averaged sensitivity (d′) and (**D**) hit reaction times. Stars indicate significant differences (n_mus_ = 12; n_n-mus_ = 12; *p* < 0.05).

**Figure 3:**
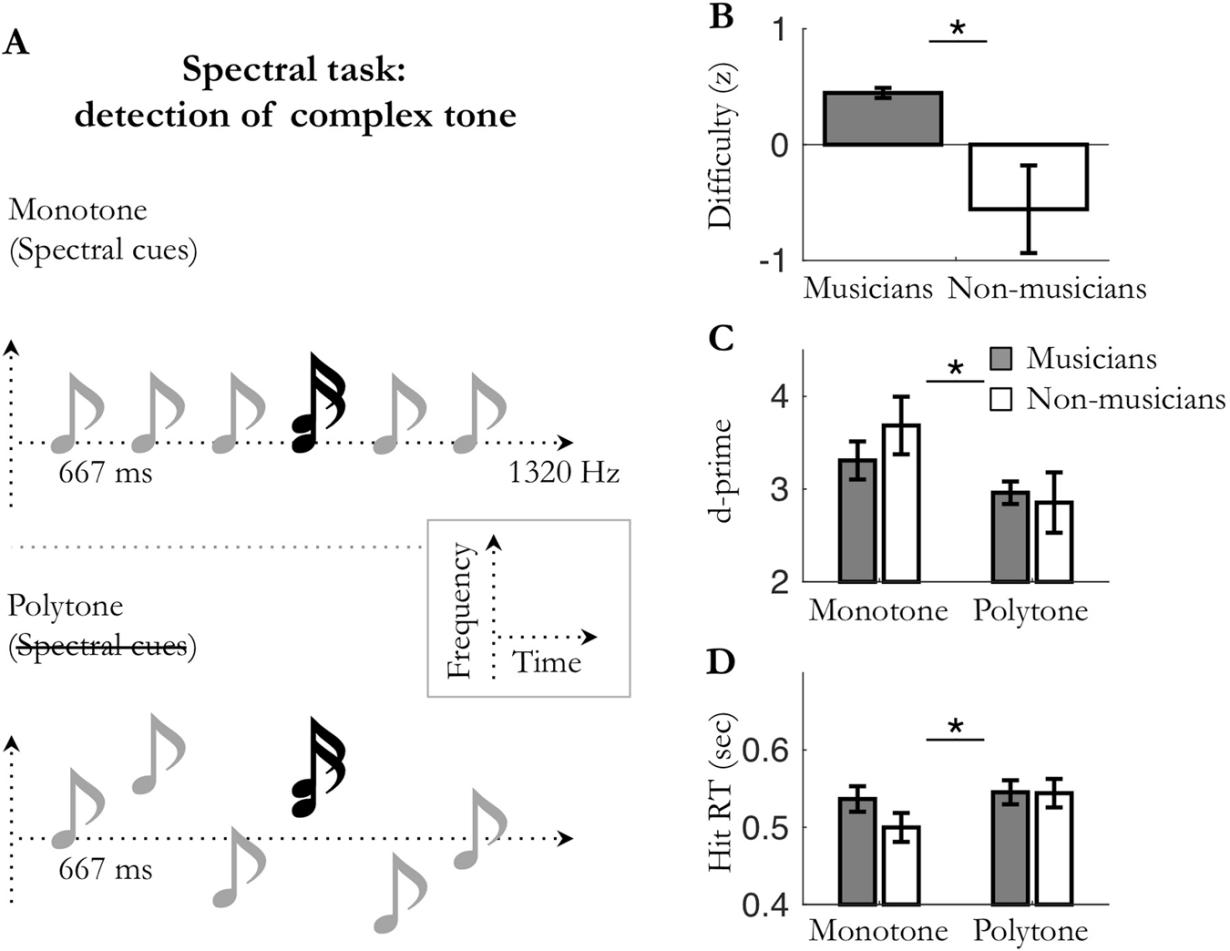
Experiment 3: spectral attention in *monotone*/*polytone* periodic conditions. (**A**) Periodic (1.5 Hz) auditory streams of pure tones were presented in the *monotone* (fixed pitch; e.g. 1320 Hz) or the *polytone* (jittered pitch) condition. Participants had to detect spectrally deviant, complex tones (dyads). (**B**) Averaged difficulty for musicians and non-musicians (set to equalize performance accuracy). (**C**) Averaged sensitivity (d′) and (**D**) hit reaction times. Stars indicate significant differences (n_mus_ = 15; n_n-mus_ = 12; *p* < 0.05).

**Figure 4:**
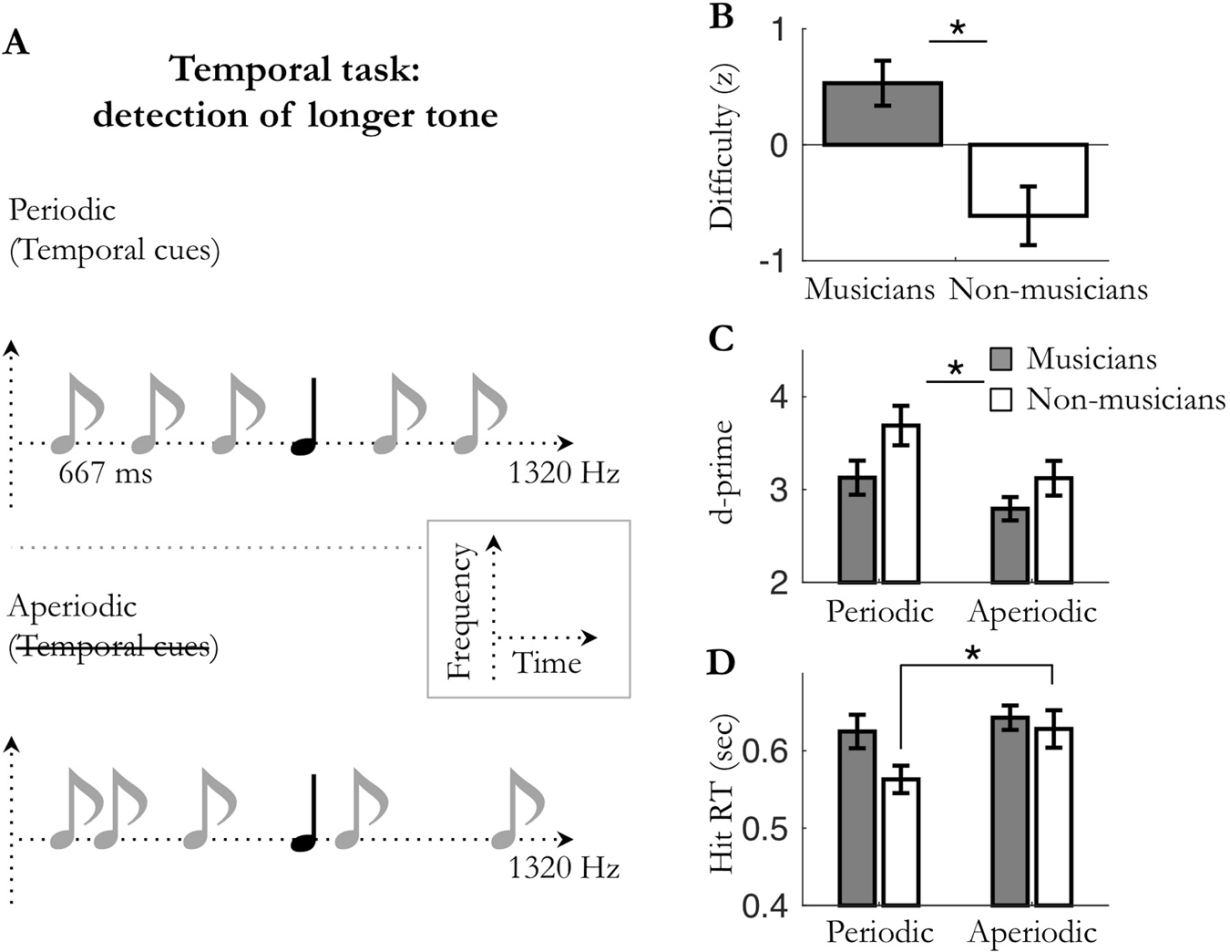
Experiment 4: temporal attention in *periodic/aperiodic* monotone conditions. (**A**) Monotone (e.g. 1320 Hz) auditory streams of pure tones were presented in the *periodic* (fixed SOA; 667 ms) or the *aperiodic* (jittered SOA) condition. Participants had to detect temporally deviant, longer tones. (**B**) Averaged difficulty for musicians and non-musicians (set to equalize performance accuracy). (**C**) Averaged sensitivity (d′) and (**D**) hit reaction times. Stars indicate significant differences (n_mus_ = 15; n_n-mus_ = 13; *p* < 0.05).

The goal of experiments 1 and 2 was to investigate whether and how predictions in one dimension (spectral or temporal) can optimize perceptual detection in the *other* dimension. In other words, we studied whether regularities in the dimension of non-interest for the task can optimize performance. Spectral predictability depended on whether the sequence had a constant pitch (monotone) or not (polytone), and temporal predictability depended on whether the sequence was regular (periodic) or not (aperiodic). In experiment 1 the spectrally deviant targets were of higher pitch than the monotone stream, and in experiment 2 the temporally deviant targets were occurring off beat from the periodic stream. The goal of experiments 3 and 4 was to investigate whether and how predictions in one dimension (spectral or temporal) can optimize perceptual detection in the *same* dimension. In other words, we studied whether regularities in the task-relevant dimension can optimize performance. To do so, we had to manipulate two different features of the task-relevant dimension. In experiment 3, while spectral predictability depended on whether the sequence was monotone or polytone, the spectral deviant targets were two-note chords. In experiment 4, while temporal predictability depended on whether the sequence was periodic or aperiodic, the temporal deviant targets were longer tones. These first four experiments thus had the following design: spectral task in periodic/aperiodic monotone conditions (exp. 1; Fig. 1a); temporal task in monotone/polytone periodic conditions (exp. 2; Fig. 2a); spectral task in monotone/polytone periodic conditions (exp. 3; Fig. 3a); and temporal task in periodic/aperiodic monotone conditions (exp. 4; Fig. 4a).

In all experiments (1–4), task difficulty was adjusted to each listener using a staircase procedure to reach 75% accuracy (exp. 1 and 2) or 50% accuracy (exp. 3 and 4) for detecting deviant targets in the least predictable condition. In experiments 1–2, tones were embedded in white noise and difficulty was set by adjusting the signal-to-noise ratio of the sequence. In experiments 3 and 4, difficulty was set by respectively adjusting the relative sound level of the targets within the dyad and the duration of the targets. Analyses revealed that the adjusted difficulty level was significantly higher for musicians than non-musicians, in all four experiments (unpaired Mann-Whitney tests: Exp. 1: *U* = 29, *p* = 0.014, *r* = 0.50; Exp. 2: *U* = 35, *p* = 0.033, *r* = 0.44; Exp. 3: *U* = 43, *p* = 0.023, *r* = 0.44; Exp. 4: *U* = 31.5, *p* = 0.002, *r* = 0.58; Figs 1–4b). Perceptual sensitivity d′ for detecting targets were subjected to a mixed factorial two-way parametric ANOVA with predictability context (monotone, polytone; or periodic, aperiodic) as within-subject factor, and musical expertise (musicians, non-musicians) as between-subject factor (Figs 1–4c). No significant main effect of musical expertise was found on perceptual sensitivity d′ in either task type (all *ps* ≥ 0.068; Figs 1–4c). Importantly, in the least predictable condition, post-hoc tests did not reveal any significant difference on d′ between groups (unpaired t-tests: all *ps* ≥ 0.14), confirming that the difficulty level was initially properly fine-tuned between musicians and non-musicians. Overall, these results reveal that musicians could perform similarly to non-musicians at higher task difficulty levels.

In all experiments, perceptual sensitivity *d*′ was significantly greater in the predictable condition than the unpredictable one (main effect of predictability: Exp. 1: F_(1,22)_ = 36.0, *p* < 0.001; Exp. 2: F_(1,22)_ = 60.2, *p* < 0.001; Exp. 3: F_(1,25)_ = 11.8, *p* = 0.002; Exp. 4: F_(1,26)_ = 23.5, *p* < 0.001; Figs 1–4c). These results indicate that sensory expectation, whether it be spectral predictions or temporal predictions, results in increased perceptual sensitivity compared to a non-predictable condition. Moreover, this holds true whatever the nature of the attentional demand required by the task (temporal attention in exp. 2 and 4 or spectral attention in exp. 1 and 3). Yet, no significant interaction between musical expertise and prediction on perceptual sensitivity d′ was found in either task type (all *ps* ≥ 0.15; Figs 1–4c). Of note, none of the observed predictions-related effects could be accounted for by a change in speed-accuracy trade-off (i.e., joint increases or decreases in sensitivity and reaction times; see supplementary results and Figs 1–4d).

Overall, these results reveal that sensory predictions, whether it be spectral predictions or temporal predictions, optimize perceptual sensitivity d′ (and reduce hit reaction times; see supplementary results) in both spectral and temporal detection tasks. Moreover, neither group benefited more of sensory predictions than the other. Importantly, this result was observed with each group adjusted to its own physiological range (*i*.*e*. with normalized difficulty level). The enhancement of perceptual detection in predictable vs. non-predictable conditions thus appear to be independent of the past knowledge of the listener.

### Perceptual sensitivity in complex predictability contexts (two-dimensional predictions)

**Figure 5:**
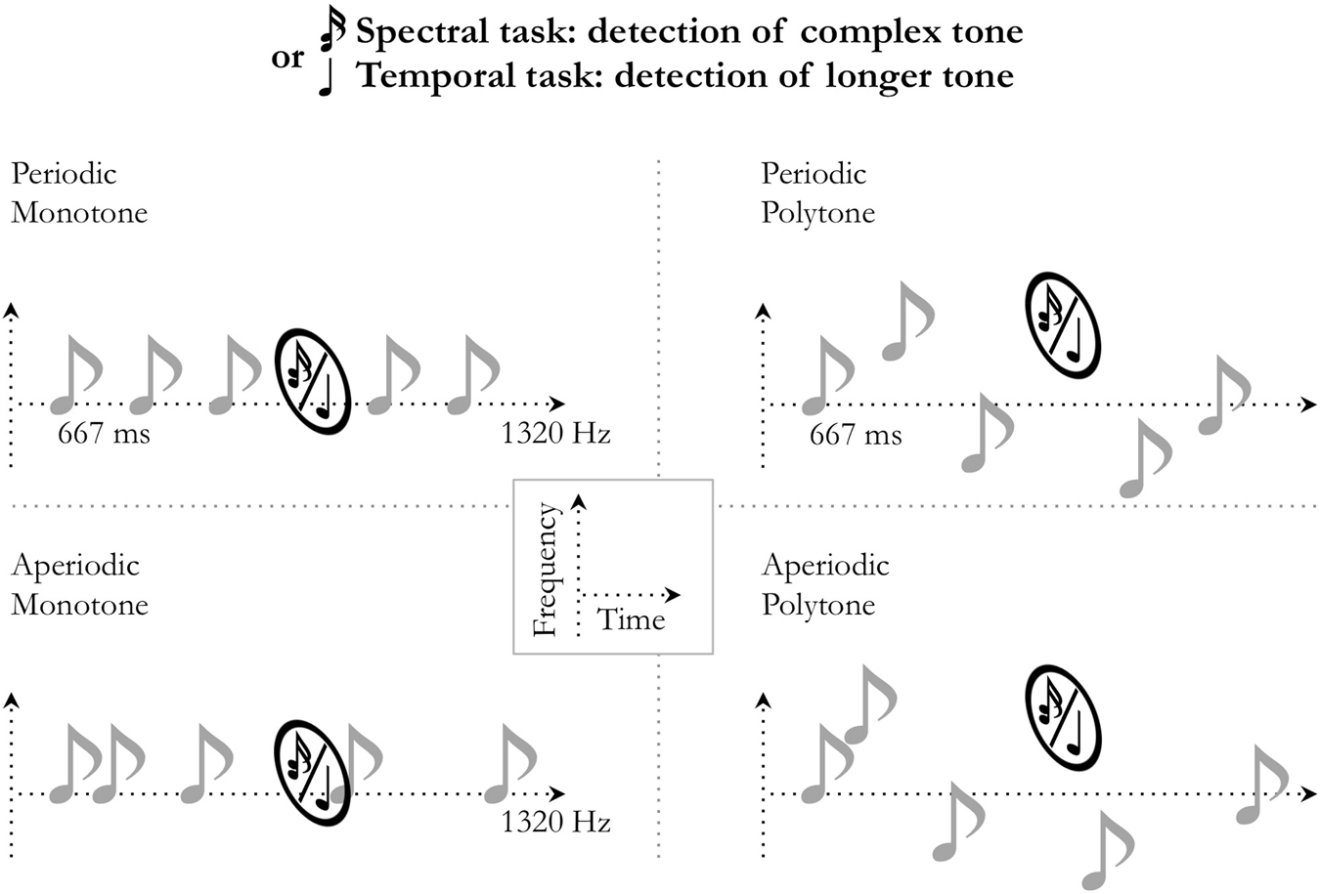
Design of experiments 5 and 6: spectral or temporal attention in periodic/aperiodic monotone/polytone conditions. Auditory streams of pure tones were presented in a *periodic* (fixed SOA; 667 ms) or *aperiodic* (jittered SOA) context, and/or *monotone* (fixed pitch; e.g. 1320 Hz) or *polytone* (jittered pitch) context. This resulted in four conditions: periodic/monotone; aperiodic/monotone; periodic/polytone; and aperiodic/polytone. Participants had to detect spectrally deviant, complex tones (dyads), or temporally deviant, longer tones.

**Figure 6:**
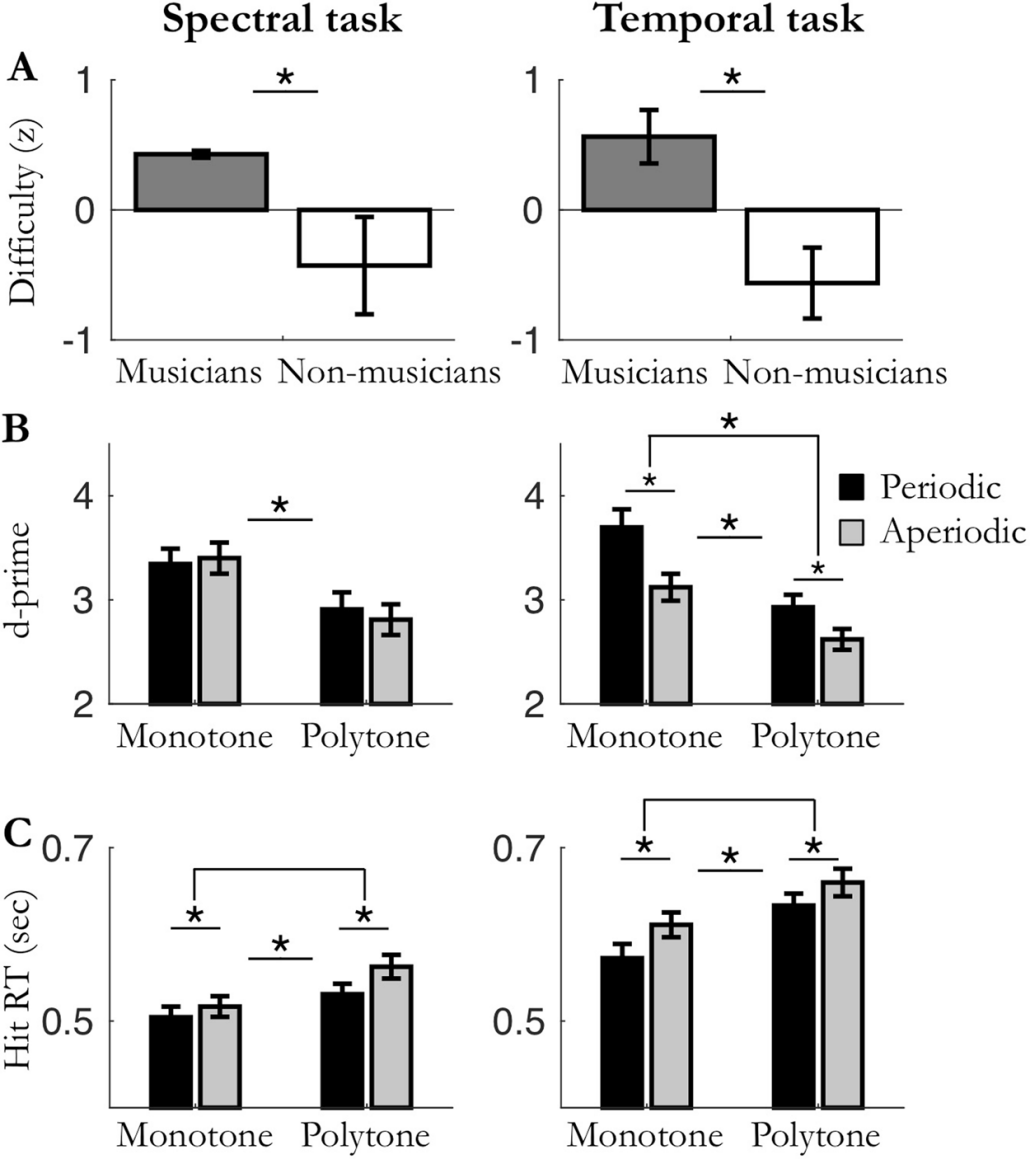
Results of experiments 5 and 6: effects of type of task and predictability contexts. (left) spectral task, (right) temporal task. (**A**) Averaged difficulty for musicians and non-musicians (set to equalize performance accuracy; n_mus_ = 12; n_n-mus_ = 12; *p* < 0.05). (**B**) Averaged sensitivity (d′) and (**C**) hit reaction times. Data from musicians and non-musicians are pooled. Stars indicate significant differences (n_mus+n-mus_ = 24; *p* < 0.05).

**Figure 7:**
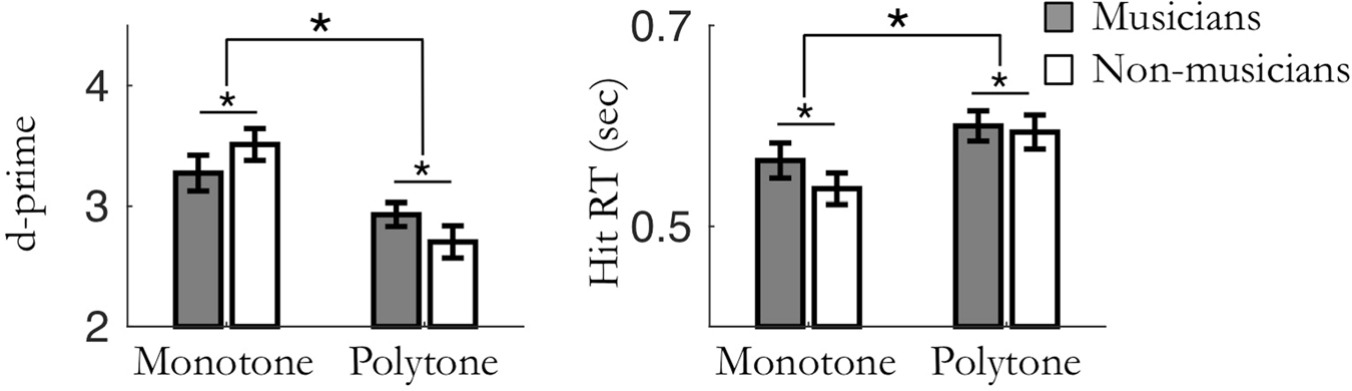
Results of experiments 5 and 6: effect of musical expertise in *monotone*/*polytone* contexts. (left) Averaged sensitivity (d′) and (right) hit reaction times. Data from the two tasks and two temporal predictability contexts are pooled. Stars indicate significant differences (n_mus_ = 12; n_n-mus_ = 12; *p* < 0.05).

Again, in experiments 5 and 6 (Fig. 5) task difficulty was adjusted to each listener to reach 50% accuracy for detecting deviant targets in the least predictable condition (here, polytone aperiodic condition). Analyses revealed that the adjusted difficulty level was significantly higher for musicians than non-musicians (unpaired Mann-Whitney tests: Exp. 5: *U* = 38, *p* = 0.053, *r* = 0.40; Exp. 6: *U* = 24, *p* = 0.006, *r* = 0.57; Fig. 6a). Perceptual sensitivity d′ for detecting targets were submitted to a mixed factorial four-way parametric ANOVA with type of task (spectral, temporal), spectral predictability context (monotone, polytone) and temporal predictability context (periodic, aperiodic) as within-subject factors, and musical expertise (musicians, non-musicians) as between-subject factor (Figs 6b and 7).

### Task-dependent synergistic combination of temporal and spectral predictions

First, the ANOVA showed that neither task was easier than the other (main effect of type of task on d′: F_(1,22)_ = 0.019, *p* = 0.89; Fig. 6b). Moreover, a post-hoc analysis indicated that in the least predictable condition (polytone aperiodic condition), d′ was not significantly different across tasks (paired t-test: t_(23)_ = 1.03, *p* = 0.31), which confirm that the difficulty level was properly fine-tuned between spectral and temporal tasks. Second, the ANOVA analysis revealed significant main effects of both types of predictability contexts on d′ (spectral: F_(1,22)_ = 59.5, *p* < 0.001; temporal F_(1,22)_ = 15.0, *p* = 0.001). Thus, both temporal and spectral predictions optimize perceptual sensitivity, but as main effects, these results do not distinguish tasks and whether sensory predictions are presented in isolation or in combination. Third, we found a significant interaction between type of task and temporal predictability context (F_(1,22)_ = 16.1, *p* = 0.001). Post-hoc tests revealed that d′ were enhanced by temporal predictions only in the temporal task (paired t-tests: temporal task: t_(23)_ = 6.07, *p* < 0.001; spectral task: t_(23)_ = 0.25, *p* = 1.0; Bonferroni corrected with a factor 2). It is of note that no significant interaction was found between type of task and spectral predictability context (F_(1,22)_ = 0.49, *p* = 0.49), or between temporal and spectral predictability contexts (F_(1,22)_ = 0.45, *p* = 0.51). This indicates that while perceptual sensitivity is enhanced by spectral predictions in both spectral and temporal tasks, temporal predictions improve sensitivity only in the temporal task. (Notice that these main interaction results do not distinguish whether sensory predictions are presented in isolation or in combination.) Finally, we found a significant triple interaction between type of task, spectral predictability context and temporal predictability context (F_(1,22)_ = 6.18, *p* = 0.021). Post-hoc tests were conducted in the temporal task only, since the main effect of temporal predictions was only observed in this task. They revealed that the effect of temporal predictions on d′ was stronger in the monotone context than in the polytone context, although significant in both contexts (paired t-tests: monotone context: t_(23)_ = 6.48, *p* < 0.001; polytone context: t_(23)_ = 3.52, *p* = 0.004; Bonferroni corrected with a factor 2). This suggests that spectral and temporal predictions synergistically combine to optimize sensitivity d′ (and reduce hit reaction times; see supplementary results and Fig. 6c) when attention is in the time domain (temporal task), but not when it is in the spectral domain (spectral task).

### Musicians and non-musicians differently exploit spectral predictions in complex contexts

The four-way ANOVA finally did not reveal a significant main effect of musical expertise on d′ (F_(1,22)_ = 0.001, *p* = 0.97, Fig. 7). Importantly, in the least predictable condition, post-hoc tests did not reveal any significant difference between groups in both type of tasks (unpaired t- tests: spectral task: t_(22)_ = 1.16, *p* = 0.26; temporal task: t_(22)_ = 0.63, *p* = 0.53), confirming that the difficulty level was initially properly fine-tuned between musicians and non-musicians. These results reveal that, as for one-dimensional predictability contexts, the musicians needed a significantly higher difficulty level to perform similarly to the non-musicians in bi-dimensional predictability contexts.

We also observed a significant interaction effect between musical expertise and spectral predictability context (F_(1,22)_ = 9.76,, p = 0.005). Post-hoc tests revealed that the effect of spectral predictions on d′ was stronger for non-musicians than musicians, although significant in both groups (paired t-tests: musicians: t_(11)_ = 4.55, *p* = 0.002; non-musicians: t_(11)_ = 6.27, *p* < 0.001; Bonferroni corrected with a factor 2). It is of note that no significant interaction was found between type of task and musical expertise (F_(1,22)_ = 0.40, *p* = 0.54), or between musical expertise and temporal predictability context (F_(1,22)_ = 0.69, *p* = 0.42). Finally, no significant other triple or quadruple interaction was found (all *ps* ≥ 0.32). Overall, this suggests that when the predictability context of the experiment is complex (i.e. both temporal and spectral contexts are jointly manipulated), non-musicians capitalize more on spectral predictions to optimize their perceptual sensitivity (and reduce their response time; see supplementary results and Fig. 7) than experts. However, the average d′ (and reaction times) did not differ between groups, and musicians had a higher adjusted difficulty level than non-musicians (see above).

## Discussion

This study was designed to assess the respective and combined influence of temporal and spectral predictions – distinct features of the same auditory stream – on perceptual sensitivity in both musical experts and non-musicians listeners. The ultimate goal of the study was to decipher the underlying organizational principles of spectrotemporal predictions in human auditory attention.

### Organizational principles of multidimensional predictions

In line with previous studies, our results first demonstrate that, on their own, temporal predictions enhance perceptual discriminability of auditory events (Figs 1 and 4)^8,14,32–34^. Importantly, we extended this finding to another auditory dimension, the spectral one (Figs 2 and 3), which points to a rather canonical form of prediction-related perceptual optimization. Moreover, these behavioral effects occurred independently of the focus of attention (spectral or temporal) required by the task, revealing that the impact of one-dimension predictions (simple predictability context) on perceptual sensitivity does not depend on behavioral relevance (i.e. task demands). The same results being found under different experimental contexts (predictions enhanced auditory sensitivity in the presence (exp. 1–2) or absence (exp. 3–4) of white noise, with task difficulty being set by adjusting the detectability of the entire auditory stream (exp. 1–2) or of only targets (exp. 3–4), and with targets being either pure (exp. 1–2, 4) or complex (exp. 3) tones), this also points to the involvement of a fundamental, robust mechanism. However, when predictions are presented in combination, that is to say when spectral and temporal predictions about upcoming auditory stimuli are jointly manipulated (Figs 5 and 6), different patterns of interaction of sensory predictions emerge depending on task demands. On the one hand, in the spectral task, only spectral predictions improved perceptual sensitivity, without this effect being modulated by the co-occurrence of temporal predictions. On the other hand, in the temporal task both spectral and temporal predictions improved perceptual sensitivity. Furthermore, they did not combine in a linear fashion, but did synergistically interact to bias auditory processing. This highlights the fact that under specific task constraints, multidimensional predictions can efficiently combine to optimize perception. In sum, the effectiveness of spectral predictions in auditory detection tasks appears to be robust and independent of attention, whereas the effectiveness of temporal predictions is conditioned by several factors, such as behavioral relevance and the co-occurrence of spectral predictions.

These results have several implications. Firstly, in line with previous studies they provide evidence for a *hierarchy of predictive filters* in sensory cortices^11,14^. Indeed, the dominance of spectral predictions in both spectral and temporal tasks indicates that spectral predictions are of primary importance in auditory processing. This interpretation is reinforced by the fact that the two groups of musical expertise did not differ *qualitatively* in their ability to exploit and combine predictions (see below). Multidimensional predictions are thus organized according to a fixed canonical model.

Secondly, concerning the organizational principles governing this hierarchy of predictive filters, our results suggest that it is organized according to physiological constraints (the topography of the auditory system) rather than attentional priorities (dynamical adjustment according to task demands). The neural mechanisms through which combined spectral and temporal predictions bias auditory perception thus seem constrained by the tonotopy of the auditory system.

Thirdly, we observed some modulatory influence on this canonical model. Because task demands impacted only the effect of temporal (but not spectral) predictions, attention would have a modulatory influence on the higher levels (here, temporal) of this hierarchy of predictive filters. This result is complemented by the fact that temporal predictions did not have a significant impact on the detection of spectral deviants in experiment 5 whereas it had in experiment 1. This apparent discrepancy could be due to multiple parameters that differ between the two experiments, such as: the difference of stream contexts, relying respectively on complex (temporal and spectral) or simple (temporal only) predictability context; the specific nature of the deviants, with respectively timbre (two-note chords) or pitch (higher pitch) deviants; the absence or presence of background white noise; or the fact that difficulty was adjusted by modulating the detectability of the targets only or of the entire auditory stream. Whichever it may be, our data points to the fact that higher-order cognitive factors such as attention, and possibly contextual factors^35^ influence the impact of temporal, but not spectral, predictions on auditory sensitivity.

### Relationship between musical expertise and the influence of predictions on perceptual sensitivity

In order to decipher the influence of predictions on perceptual sensitivity we needed to place our investigations in psychophysical regimes that allow fluctuations in performance accuracy across conditions (i.e. to avoid ceiling or floor effects). In all experiments, difficulty levels were thus normalized for each participant, such that both groups performed the tasks in their respective psychophysical regime. In this respect, our results provide the first evidence that there is no *qualitative* difference between musicians and non-musicians in their ability to use one-dimensional (spectral or temporal, Figs 1–4) or bi-dimensional (spectrotemporal, Fig. 7) predictions to improve auditory perception. However, in all experiments there was an influence of musical expertise on the general difficulty level settings. Musicians performed better than non-musicians in the least predictable condition (absence of temporal and/or spectral regularities depending on the experiment). Then, adding contextual regularities benefited performance in both groups in a similar fashion, that is to say according to the same organizational principles (see above). In one-dimensional predictability contexts (Figs 1–4), there was indeed no quantitative difference of prediction gain (the difference of performance between predictable and unpredictable conditions) between groups. In bi-dimensional (complex) predictability contexts, the only significant quantitative difference was for spectral prediction gain, with non-musicians capitalizing more on spectral cues than experts to optimize their perceptual sensitivity. Overall, these findings clearly suggest that the same perceptual mechanisms underlie the processing of predictions in musical experts and non-musicians; long-term musical expertise would then have a subtle quantitative impact on the way sensory predictions modulate perception, operationalized in complex contexts by a lesser emphasis on spectral predictions.

These findings have three major implications. First, the fact that non-musicians capitalized more on spectral predictions in complex auditory scenes (Fig. 7) suggests that predictive information about the spectral content of upcoming stimuli is inherently more easily exploitable by non-experts than temporal predictions, which is in accordance with the proposal of a tonotopically-based hierarchy of predictive filters.

The second point concerns the putative musician enhancements reported in the literature. Our results indeed reveal that the perceptual advantage induced by musical expertise commonly observed in perceptual tasks (e.g. in rhythm information perception^36,37^, or spectral information perception^38–40^) stems from the difference of operating regimes between musicians and non-musicians. In our tasks, the fact that musicians outperform non-musicians in the least predictable condition likely reflects that musicians are less susceptible to informational masking, when using both masking noise (Figs 1 and 2) and masking tones (Figs 3 and 6), also meaning that they are better in tone-in-noise perception^41,42^. This also reflects that musicians have enhanced auditory short-term memory^43,44^, and, as other perceptual experts, have better auditory sustained attention for complex tone patterns^25,45^. In other words, our results provide new evidence that musical expertise impacts basic sound representation and higher-level cognitive factors subtending the detection of perceptual deviants^26,27^. These benefits allegedly lead to improved abilities to detect regularities, that is to say to *generate* predictions within any acoustic environment^29,46^. However, our results also show that musical expertise does not increase prediction gain, which points to the fact that non-musicians’ ability to exploit explicit (i.e. easily detectable) regularities is quite similar to musicians. Our study thus emphasizes the distinction between the capabilities of *generating* predictions from those of *exploiting* (already- or) easily-formed predictions. In this respect, the present results clearly suggest that the putative musicians enhancements rely on a better ability to generate predictions, that is to say to detect subtle regularities within the environment in order to form internal models, but not to exploit predictions, i.e. to use current internal models to optimize perception. Overall, this suggests that perceptual learning differentially impact complementary mechanisms or pathways^47^ underlying auditory perception in a volatile environment.

Third, theoretically perceptual expertise could impact performance accuracy in two complementary ways, either by increasing the sensitivity to regularities in the environment (i.e. contrast gain mechanism; this means that experts’ performances are similar to that of non-experts with less contextual regularity^48^), or by increasing the impact that any given contextual regularity has on perceptual sensitivity (i.e. response gain mechanism; this means that the more there is contextual regularity, the more experts outperform non-experts). In our study, by contrasting performance in contexts of maximal and minimal regularity, we could only address the second hypothesis, i.e. whether the impact of perceptual expertise on performance accuracy is supported by a response gain mechanism. Our results demonstrate that the impact of musical expertise is similar in both maximal and minimal regularity contexts; expertise does not increase prediction gain and is thus not supported by a response gain mechanism. This suggests that the putative impact of musical expertise on auditory perception is supported by a contrast gain mechanism, a better ability to generate predictions (i.e. an increased sensitivity to regularities in the environment).

## Conclusion

In conclusion, our study points to a hierarchical organization of predictions that is consistent with physiological constraints imposed by the topography of the sensory system of interest. Nevertheless, musical expertise and attention seem to have a secondary modulatory influence, strongly dependent on the complexity of the perceived sensory stream, with more complexity enabling more modulatory influence.

## Method

This study investigates the relationship between attention (task relevance) and prediction (signal probability) along two dimensions of auditory stimuli: frequency and time. It includes six experiments (see below).

### Participants

All participants tested in this set of experiments reported normal hearing and had no history of audiological or neurological disorders. All methods were performed in accordance with the relevant guidelines and regulations. All experimental protocols were approved by the Local Ethics Committee (CPP Méditerranée Sud, A01490-49). Participants gave their informed consent and were paid for their participation. In all experiments, participants were subdivided into two groups based on their musical expertise: the group of musician listeners (semi-professional or professional musicians) and the group of non-musician listeners (participants with less than 2 years of formal musical training, and not currently engaged in active music making). We did not take amateur musicians in these experiments.

*Experiment* 1 comprised 24 volunteers: 12 musicians (3 females; mean age: 29.3 ± 4.7 yr) and 12 non-musicians (3 females; mean age, 34 ± 16.2 yr). *Experiment* 2 comprised 24 volunteers: 12 musicians (6 females; mean age, 30.8 ± 9.4 yr) and 12 non-musicians (8 females; mean age, 30.7 ± 11.5 yr). *Experiment* 3 comprised 27 volunteers: 15 musicians (9 females; mean age, 28.5 ± 8.6 yr) and 12 non-musicians (5 females; mean age, 29.2 ± 7.6 yr). *Experiment* 4 comprised 28 volunteers: 15 musicians (9 females; mean age, 26.9 ± 7.8 yr) and 13 non-musicians (5 females; mean age, 31.9 ± 9.2 yr). *Experiments* 5 *and* 6 comprised 24 volunteers: 12 musicians (8 females; mean age, 27.5 ± 7.9 yr) and 12 non-musicians (4 females; mean age, 29.2 ± 7.5 yr).

### Apparatus

Auditory stimuli were generated and presented to the participants at a sampling rate of 44.1 kHz with 16-bit resolution, using Matlab (MathWorks) and the Psychophysics Toolbox extensions. Sounds were converted using a RME Fireface 800 soundcard, amplified using a Grace m904 (Grace Design) and presented diotically through headphones (Beyerdynamic DT 770 PRO). Sound levels were calibrated using a Brüel & Kjær artificial ear (type 4153) and microphone (type 4191) coupled with the mounting plate provided for circumaural headphones.

### Tasks and design

Experiments 1, 3, and 5 required spectral attention (i.e. detection of rule-violating stimuli in the frequency domain), and experiments 2, 4, and 6 required temporal attention (i.e. detection of rule-violating stimuli in the time domain).

Each trial consisted of an auditory stream of pure tones (100-ms long, dampening length 10 ms and attenuation 40 dB) lasting approximately one minute. This comprised a majority of standard stimuli interleaved with 6 (exp. 1–2) or 10 (exp. 4–6) target stimuli, the so-called deviants (see below, Targets section), inserted pseudo-randomly in the stream, every 3 to 12 seconds. Participants carried out these deviant detection tasks in different contexts of temporal and/or spectral predictability. In experiments 1 and 2 predictability was modulated in the non-attended dimension (i.e. temporal predictability in exp. 1, spectral predictability in exp. 2). In experiments 3 and 4 predictability was modulated in the attended dimension. In experiments 5 and 6 predictability was modulated in both the attended and non-attended dimensions. Predictability was always modulated in a binary manner (predictable/unpredictable condition), resulting in 2 conditions in experiments 1–4 (presence/absence of temporal or spectral predictions), and 4 conditions in experiments 5–6 (presence/absence of temporal and/or spectral predictions). Of note, in experiments 1–4 the non-manipulated dimension was always predictable.

Temporal predictability depended on whether the sequence was rhythmic or not. It was manipulated by varying the regularity of the stimulus-onset asynchrony (SOA), centered around 667 ms (1.5 Hz). In *periodic* conditions, the SOA was quasi-fixed, with a residual jitter of ±5% (sampled from a uniform distribution). In *aperiodic* conditions (exp. 1 and 4–6), the SOA was jittered of ±30% (exp. 1) or ±60% (exp. 4–6). Spectral predictability depended on whether the sequence had a constant pitch or not. In *monotone* conditions, the pitch was quasi-fixed within a trial, with a residual jitter of ±½ semitone. In *polytone* conditions (exp. 2–3 and 5–6), the pitch was centered around 880 Hz (exp. 2) or 1320 Hz (exp. 3 and 5–6) and was jittered within a range of one octave above and below the center frequency. Of note, in the *monotone* condition the pitch varied across trials, so that the frequency distribution of the tones was on average matched between *monotone* and *polytone* conditions.

The six experiments had thus the following design: spectral attention in periodic/aperiodic monotone conditions (exp. 1); temporal attention in monotone/polytone periodic conditions (exp. 2); spectral attention in monotone/polytone periodic conditions (exp. 3); temporal attention in periodic/aperiodic monotone conditions (exp. 4); spectral attention in periodic/aperiodic monotone/polytone conditions (exp. 5); and temporal attention in periodic/aperiodic monotone/polytone conditions (exp. 6).

### Targets

In experiments 1–2, tones were presented near-threshold, embedded in white noise. The noise level was set to 70 dBA SPL. Difficulty was set by adjusting the signal-to-noise ratio (respectively −19 dB and −18 dB on average), i.e. individually tailoring the global detectability of the auditory stream (see Procedure section). In experiment 1, the spectrally deviant targets were of higher pitch than the monotone stream (3 semitones). In experiment 2, the temporally deviant targets were occurring off beat from the periodic stream (+400 ms relative to the 1.5 Hz beat, i.e. 667 ms period). This manipulation ensured that the putative impact of predictive mechanisms over attentional ones was maximized^49^. In addition, it is of note that over the frequency range under study ([440 2640] Hz), pure tones detection thresholds in the presence of white noise are virtually uniform^50,51^, such that target and standard tones presented similar perceptual emergence.

In experiments 3–6, tones were presented at a comfortable listening level (~60 dB SPL), and were not embedded in white noise. Difficulty was set by individually tailoring the detectability of the target tones (see Procedure). In experiments 3 and 5, the spectrally deviant targets were co-occurring with a standard tone and were of higher pitch (3 semitones). This resulted in the percept of two-note chords (dyads). The global sound level of the dyad was normalized with the standard tones. Difficulty was set by adjusting the relative sound level of the targets within the dyad (9% of the dyad level on average). In experiments 4 and 6, the temporally deviant targets were of longer duration. Difficulty was set by adjusting the duration of the targets (142 ms on average, with standard stimuli lasting 100 ms).

### Procedure

The procedure was identical across all experiments. At the start of the session, task difficulty was individually tailored using an adaptive psychophysical staircase procedure to estimate the threshold level for detecting deviant tones with 75% (exp. 1–2) or 50% (exp. 3–6) hit rate accuracy (these threshold levels were set based on pilot data to avoid floor or ceiling d′ effects in the experiment). The calibration was performed on 10 trials in the unpredictable condition (i.e. aperiodic or/and polytone condition). Following this preliminary calibration phase, which also served as a familiarization procedure, participants started the proper experiment. Conditions were presented in a randomized order. Participants were instructed to press a button as soon as they heard a deviant tone in the stream of auditory stimuli. Feedback was provided at the end of each trial indicating the number of correct responses and the number of false alarms. Each experiment lasted approximately one hour, for a total of 168 (exp. 1–2), 100 (exp. 3–4), or 80 (exp. 5–6) deviants per condition.

### Analyses procedures

Across experiments, task difficulty was fine-tuned by adjusting different parameters, such as the signal-to-noise ratio of the sequence (exp. 1–2), the relative sound level of the targets within the dyad (exp. 3 and 5) and the duration of the targets (exp. 4 and 6). The opposite of these values directly indexes difficulty (as smaller values mean higher difficulty). For each experiment, we then z-scored this latter value across participants (musicians and non-musicians) to obtain a normalized difficulty index across experiments. Responses with a reaction time within the range [0.25 1.2] s following targets’ onsets were counted as hits, and other responses were considered to be false alarms. Participants’ responses were transformed into sensitivity index (d′) and reported, together with hit reaction times, as mean ± standard error. Analyses were performed at the single-subject level before applying standard parametric or non-parametric two-sided statistical tests at the group level in SPSS ((unpaired) Mann-Whitney signed-rank tests or mixed factorial ANOVAs) and statistical significance thresholds were set at p < 0.05. For t-tests analyses on task difficulty measures we applied the non-parametric version of the test as for most of our datasets (exp. 2–5), the data residuals were significantly deviating from the normal distribution (Shapiro-Wilk normality test: *p* < 0.05). For d′ and correct RT measures, we applied parametric mixed design ANOVAs (with parametric post-hoc t-tests), as non-parametric tests are less standard to estimate significance for interaction terms, and parametric F tests are robust to non-normality to some extent.

## Electronic supplementary material

Supplementary results
