# Supplementary material for: Organizational principles of multidimensional predictions in human auditory attention
Source: Sci Rep. 2018 Sep 7;8:13466. doi: 10.1038/s41598-018-31878-5 (PMC6128843; doi:10.1038/s41598-018-31878-5)
Supplement: Supplementary file 1 — Supplementary results [file 41598_2018_31878_MOESM1_ESM.docx]

**Organizational principles of multidimensional predictions in human auditory attention**

**Indiana Wollman^1, 2^ and Benjamin Morillon^3^**

^1^ Montreal Neurological Institute, McGill University, Montreal, Canada

^2^ CIRMMT, Schulich School of Music, McGill University, Montreal, Canada

^3^ Aix Marseille Univ, Inserm, INS, Inst Neurosci Syst, Marseille, France

**Supplementary results: hit reaction times (RTs)**

In experiments 1-4, hit RTs were submitted to a mixed factorial two-way parametric ANOVA with predictability context (monotone, polytone; or periodic, aperiodic) as within-subject factor, and musical expertise (musicians, non-musicians) as between-subject factor (Fig. 1-4d). In all experiments, RTs were significantly shorter in the predictable than unpredictable condition (main effect of predictability: Exp. 1: F_(1,22)_ = 21.8, *p* < .001; Exp. 2: F_(1,22)_ = 153.0, *p* < .001; Exp. 3: F_(1,25)_ = 8.09, *p* = .009; Exp. 4: F_(1,26)_ = 22.9, *p* < .001). These results confirm that sensory expectation, whether it be spectral predictions or temporal predictions, results in optimized performance (i.e. increased sensitivity d’ as well as reduced RTs) compared to a non-predictable condition. Additionally, a significant main effect of musical expertise was found on hit reaction times (RTs) in experiment 2 (Exp. 2: F_(1,22)_ = 5.41, *p* = .030; Exp. 1,3,4: all *ps* ≥ .17), and a significant interaction between musical expertise and predictability context on RTs was found in experiment 4 (Exp. 4: F_(1,22)_ = 7.42, *p* = .011; Exp. 1-3: all *ps* ≥ .068). Post-hoc analyses indicated that in this experiment the effect of predictions on RTs was beneficial for non-musicians only (paired t-tests: non musicians: t_(12)_ = 6.93, *p* < .001; musicians: t_(14)_ = 1.28, *p* = .44; Bonferroni corrected, with a factor 2; Fig. 4d), and was not accompanied by a modulation of d’. Overall, in experiments 1-4, spectral and temporal sensory predictions augment perceptual sensitivity d’ and reduce hit reaction times (RTs) in both spectral and temporal detection tasks (Fig. 1-4c-d). Moreover, neither group benefited more of sensory predictions than the other.

In experiments 5-6, hit RTs were submitted to a mixed factorial four-way parametric ANOVA with type of task (spectral, temporal), spectral predictability context (monotone, polytone) and temporal predictability context (periodic, aperiodic) as within-subject factors, and musical expertise (musicians, non-musicians) as between-subject factor (Fig. 6c and 7). The aim of this analysis was to control that none of the observed d’ results could be accounted for by a change in speed-accuracy trade-off (i.e., joint increases or decreases in sensitivity and reaction times). Therefore, we only considered the main effects of the ANOVA on the hit RTs, but for the sake of completeness we provide also below the statistics of the interactions. First, a main effect of task was observed, revealing that RTs were shorter in the spectral task (F_(1,22)_ = 70.9, *p* < .001). Second, as for d’, the analysis revealed significant main effects of both types of predictability contexts on RTs (spectral: F_(1,22)_ = 80.7, *p* < .001; temporal F_(1,22)_ = 48.4, *p* < .001). Of note, as for d’, no main effect of musical expertise was observed (F_(1,22)_ = 0.57, *p* = .46). Third, a significant interaction between musical expertise and spectral predictability context was observed, as for d’, (F_(1,22)_ = 4.68, p = .042), and no significant interactions were observed between type of task and spectral or temporal predictability contexts, spectral and temporal predictability contexts, or musical expertise and temporal predictability context (all *ps* ≥ .11). However, and contrary to d’, a significant interaction was found between type of task and musical expertise (F_(1,22)_ = 7.28, p = .013). Moreover, two significant triple interactions were found between type of task, spectral predictability context and temporal predictability context, as for d’ (F_(1,22)_ = 5.70, p = .026), and between type of task, spectral predictability context and musical expertise (F_(1,22)_ = 5.45, p = .029; other triple interactions, *ps*  ≥ .083). Finally, the quadruple interaction was significant (F_(1,22)_ = 11.4, p = .003). Overall, results on d’ and RTs suggest that spectral and temporal predictions synergistically combine to optimize performance when attention is in the time domain (temporal task), but not when it is in the spectral domain (spectral task). Moreover, a specific effect of type of task was observed on RTs but not on d’, indicating that participants were faster in the spectral than temporal task, even if their difficulty level was similar.
